# The Evaluation of Proanthocyanidins/Chitosan/Lecithin Microspheres as Sustained Drug Delivery System

**DOI:** 10.1155/2018/9073420

**Published:** 2018-07-24

**Authors:** Hong-Li Yu, Zhan-Qin Feng, Jing-Jing Zhang, Yong-Hong Wang, De-Jun Ding, Yuan-Yuan Gao, Wei-Fen Zhang

**Affiliations:** ^1^College of Pharmacy, Weifang Medical University, Weifang 261053, Shandong, China; ^2^Weifang Maternity and Child Care Hospital, Weifang 261053, Shandong, China; ^3^College of Basic Medical, Qingdao Binhai University, Qingdao 266555, Shandong, China; ^4^Collaborative Innovation Center for Target Drug Delivery System, Weifang Medical University, Weifang 261053, Shandong, China

## Abstract

Proanthocyanidin (PC) has attracted wide attention on cosmetics and pharmaceutical due to its antioxidant, anticancer, antimicrobial, antiangiogenic, and anti-inflammatory activities. However, PC applications are limited because of its sensitivity to thermal treatment, light, and oxidation and the poor absorption in the gastrointestinal tract. Thus, a novel dosage form of PC needs to be designed to improve its stability and bioavailability for drug delivery. The objective of this study is to fabricate proanthocyanidins/chitosan/lecithin (PC/CTS/LEC) microspheres and investigate various characteristics. In the current study, PC/CTS/LEC microspheres were prepared by spray-drying technology. The yield (61.68%), encapsulation efficiency (68.19%), and drug loading capacity (17.05%) were found in the results. The scanning electron microscope demonstrated that the microspheres were spherical in shape with wrinkled surfaces. DSC study displayed that the microspheres stability was greatly improved when comparing with bare PC. The in vitro release study showed that the 76.92% of PC was released from microspheres within 48 h. The moisture contents of microspheres ranged from 8% to 13%. The swelling rate and tapped density of microspheres were elevated with increasing the concentration of chitosan in the formulations. The moisture uptake of microspheres was saturated at 40°C/RH75% within 12 h. Our results indicated that the stability of PC/CTS/LEC microspheres was enhanced, and it is a promising carrier for sustained drug delivery system.

## 1. Introduction

Proanthocyanidin (PC), abundant in vegetables and fruits, is a complex mixture of catechin, epicatechin, and gallic acid esters [1]. PC is a strong natural antioxidant, containing multiple hydroxyl groups, and has attracted a considerable research interest in cosmetics and pharmaceutical preparations [2, 3]. In the cosmetics industry, PC is known to have potent antiaging, antiultraviolet, and resisting radiation capacities and whitening and moisturizing function. Moreover, PC has been extensively investigated and mainly attracted attention due to its numerous pharmacological properties, including antioxidant [4], anticancer [5], antimicrobial [6], antiangiogenic [7], and anti-inflammatory actions [8]. It has been reported that PC, even in high doses, is noncarcinogenic and nonteratogenic [9, 10]. Therefore, it is a good candidate to be a medication being applied in the biomedical field.

However, the application of PC is limited due to its sensitivity to thermal treatment, light, metallic ions, enzymes, and oxygen. Additionally, PC is poorly absorbed in the gastrointestinal tract, which compromises its bioavailability [11]. All these factors restrict its application, and a novel drug delivery system is necessary for the improvement of its stability and absorption. Many publications have discussed that the delivery of the encapsulated PC in a controlled/sustained mode might facilitate their biological activity. Huang et al. [12] reported that PC could promote drug loading and keep the drug release rate constant, and these properties made the PC-cross-linked gelatin nanofibers a perfect material for drug delivery. Cocoa procyanidins- (CPs-) gelatin-chitosan nanoparticles can enhance the stability and absorption ability of PC, which is expected to significantly heighten its biological activity. These results showed that CPs-gelatin-chitosan nanoparticles had the same apoptotic effect in human acute monocytic leukemia THP-1 cells compared with CPs in solution [13]. Our previous study revealed that oligomeric proanthocyanidins/Bletilla striata polysaccharide/chitosan (OPC/BSP/CTS) microspheres showed pronounced antioxidant activity than pure OPC [14]. By encapsulating these OPCs into biodegradable polymer bioadhesive microspheres, the deficiencies of proanthocyanidins that are easily oxidized in the air and exhibit optical instability can be overcome, and the bioavailability can be further enhanced [15]. Microspheres, serving as a carrier, can overcome disadvantages of PC when being applied in a pulmonary drug delivery system [16–18].

Chitosan (CTS), gelatin, cyclodextrins, and starch are usually used as carriers in microspheres, with CTS more often being used for this purpose [18–20]. CTS, a cationic natural biomaterial, obtained from the deacetylation of chitin, has been widely proposed as an inhalation drug carrier, for its low toxicity, biocompatibility, and biodegradability. Previous study reported that CTS can bind the mucosal surfaces because of its cationic nature, lead to a bioadhesion, and reduce mucociliary clearance, thereby providing a prolonged contact time for drugs [21]. Therefore, CTS is able to improve the drug absorption by opening the intercellular tight junctions of the lung epithelium and enhance the dissolution rate of drugs. In addition, in our previous large-scale experiments, it was found that CTS was used for the preparation of sustained release forms of pulmonary delivery microspheres due to its good biological properties, and CTS, as excipients, also had characteristics that improve the pharmaceutical and biopharmaceutical properties of drugs. Wang et al. [22] indicated that the theophylline/chitosan/*β*-cyclodextrin microspheres had a good degradation both* in vitro* and* in vivo* and could be used as a pulmonary drug delivery carrier. Zhang et al. [21] successfully prepared theophylline/chitosan/*β*-cyclodextrin microspheres, which possessed high good biocompatibility and could be applied in pulmonary sustained release systems.

Lecithin (LEC) is a lipoid compound containing phosphoric acid, a basic component of cellular biomembrane, and possesses high nutritive value. In medical and pharmaceutical industry, LEC is used not only as a drug but also as a surfactant in replace of traditional span-80 or tween-80. Furthermore, LEC is a typical amphiphilic phospholipid with good biocompatibility and capability of mixing with polymers [23]. It has been applied to enhance the hydrophilicity and biocompatibility of plain CTS materials. According to our previous study, LEC and CTS can accelerate each other's absorption rate when used in combination, resulting in a sustained therapeutic effect [24]. Therefore, we intend to develop LEC/CTS microspheres that are considered to have great application potential because it solves many drug delivery problems such as short half-life, poor bioavailability, and easy disintegration of PC. Furthermore, LEC can be applied to CTS to improve the hydrophilicity of the microspheres when the LEC was added as the pharmaceutical excipients. Accordingly, LEC has a promising future in medical and pharmaceutical fields.

In this study, we prepared PC/CTS/LEC microspheres by using spray-drying method with different drug-to-polymer ratios and investigated the characteristics of PC/CTS/LEC microspheres including the yield (YD), encapsulation efficiency (EE), drug load (DL), morphology, structures, thermal stability,* in vitro* release, moisture contents, swelling rate, tapped density, and moisture uptake. The purpose of this study was to examine the feasibility of applying the PC/CTS/LEC microspheres as a carrier for sustained release and delivery of pulmonary drugs.

## 2. Materials and Methods

### 2.1. Materials

PC (proanthocyanidins) was kindly supplied by ZeLang Group (Nanjing, China). CTS (DAC = 96.1%) was obtained from Hai Debei Marine Biotechnology Company (Jinan, China). LEC (lecithin) was purchased from the Company of Huaqing Meiheng Natural Product Technical Development (Beijing, China). Sodium Dihydrogen Phosphate and Disodium Hydrogen Phosphate were obtained from Sigma (St. Louis, MO, USA, AR).

### 2.2. Preparation of PC/CTS/LEC Microspheres

Predetermined amount of PC, LEC, and CTS was dissolved in 1000 mL of 0.5% acetic acid (Table 1). The solution was filtrated by using 0.45 *μ*m micropore film and then spray-dried with a spray dryer (Büchi® Mini Spray Dryer, B-290, Switzerland), with inlet temperature of 110°C, outlet temperature of 72~76°C, feed flow rate of 3 mL/min, and drying air flow of 400 L/h. The spray-dried microspheres were collected and stored at room temperature in desiccators (with anhydrous CaCl_2_).

### 2.3. Yield, Encapsulation Efficiency, and Drug Load

The spray-dried microspheres were collected and the YD account as the following:

(1) $$\begin{array}{c} YD\left(\;\%\;\right)=\frac{C_{M}}{C_{S}}\times 100\% \end{array}$$

where C_M_ represents the total mass of microspheres and C_S_ represents the quantity of the total added solid components.

The drug contents of the microspheres were determined using a UV (UV-1700, Shimadzu, Japan) spectrophotometer at 286 nm. An accurately amount of microspheres (an equivalent of 12.5 mg of PC) with different ratios of drug carrier (1  : 3  : 2, 1  : 1  : 1, 1  : 2  : 1, 1  : 1  : 2, 1  : 4  : 1) was crushed in a mortar with 5 mL of phosphate buffer solution (PBS, pH 7.0). PBS (pH 6.8) was used to regulate the volume of the solution to the volume of 25 mL. The ultrasonic treatment was 3 min, and it was extracted for 3 hours under the intense oscillation, which was sufficient to ensure the complete release of PC. Subsequently, the solution was filtered through a micropore film with an average pore diameter of 0.45 *µ*m. The concentration was calculated using the following standard curve equation:* y* =* 0.1438x-0.1068,* with correlation coefficient r = 0.9999 (n = 3), where* x* referred to the concentration of PC and *y* referred to the absorbance value. EE and DL were calculated using the equations as follows:

(2) EE%=CECT×100%

(3) DL%=CDCM×100%

where both C_E_ and C_D_ refer to the weight of the PC encapsulated in the microspheres, C_T_ is total PC mass added to the preparation, and C_M_ is total mass of microspheres.

### 2.4. Morphology of Microspheres

The surface morphologies of the spray-dried PC/CTS/LEC microspheres were observed by SEM (s4500n, Hitachi High-tech Science Corporation, Japan) at 20 kV. Microspheres were mounted on metal stubs using double-sided adhesive tape and sprayed coating with gold.

### 2.5. FT-IR Spectroscopy

FT-IR measurements were carried out using FT-IR Avatar-360 spectrometer (Perkin-Elmer, Japan). The samples were prepared by grinding the dry blend microspheres (2 mg) with KBr powders (100 mg) and then compressing the mixtures to form disks. Data were saved in the range of 4000~400 cm^−1^.

### 2.6. DSC Study

DSC analyses of LEC, CTS, PC, and PC/CTS/LEC were carried out using DSC7020 (Hitachi High-tech Science Corporation, Japan). Sample (5 mg) was placed onto a standard aluminum pan, crimped, and heated from 40°C to 600°C at a rate of 5°C/min with continuous purging of nitrogen (20 mL/min). An empty sealed pan was used as reference. All samples were run in triplicate.

### 2.7. In Vitro Release Studies

The release of the drug loaded PC from microspheres of different drug-to-polymer ratios was studied by using dialysis method in PBS (pH 6.8). Microspheres equivalent to 25 mg of PC were placed in dialysis bag (MWCO: 3500) with 2 mL PBS (pH 6.8) and then sealed at both ends. The dialysis bag was dipped into conical flasks containing 200 mL of release medium (PBS, pH 6.8). The conical flasks were sealed to prevent evaporation of the release medium. The whole apparatus was then placed in a shaker and was shaking at 100 rpm and maintained at 37 ± 0.5°C. 2 mL samples were withdrawn at set time intervals (15 min, 30 min, 45 min, 1 h, 1.5 h, 2 h, 3 h, 4 h, 6 h, 8 h, 12 h, 24 h, and 48 h), and the same volume was replaced with fresh PBS (pH 6.8). The samples were measured by UV spectrophotometry at 286 nm and the concentration was calculated based on the standard curve equation. The cumulative releases (CR) of PC/CTS/LEC microspheres were calculated using the following equations:

(4) $$\begin{array}{c} CR\left(\;\%\;\right)=\overset{t=\infty }{\underset{t=0}{\sum }}\frac{M_{t}}{M_{0}}\times 100\% \end{array}$$

where M_t_ refers to the cumulative releases of PC/CTS/LEC microspheres in PBS (pH 6.8) at set time intervals and M_0_ refers to the drug contents of PC/CTS/LEC microspheres.

### 2.8. Determination of Moisture Uptake

Humidity was evaluated by using the hygroscopic method. About 100 mg microspheres (W_d_) were dried to a constant weight in vacuum before being used, and they were packed into each opened weighing bottles, with three empty weighing bottles serving as blanks and then stored in the desiccators at 40°C and at a relative humidity of 75%. At the different predetermined intervals (0.5, 1, 2, 4, 6, 8, 12, 24, 48 h), the weight of the microspheres (W_h_) was recorded. The increase in weight represented the weight of moisture taken by the microspheres. All samples were analyzed in triplicate. The moisture uptake was monitored as a ratio of the weight of absorbed moisture to the weight of the dry microspheres at each period of time as follows:

(5) $$\begin{array}{c} Moisture\;\;uptake\left(\;\%\;\right)=\frac{W_{h}-W_{d}}{W_{h}}\times 100\% \end{array}$$

### 2.9. Swelling Studies

Equilibrium water uptake of the microspheres was determined by measuring the extent of swelling in PBS solutions (pH 6.8) according to the Chinese Pharmacopoeia 2010 at 37°C. Accurately weighed amounts of spray-dried microspheres (Wd, ranging from 5 to 10 mg) were dispersed in 5 mL test tubes containing 4 mL of PBS (pH 6.8). For ensuring complete equilibration, samples were allowed to swell for 24 h to obtain equilibrium. The microspheres were removed from the swelling medium and blotted with filter paper to absorb water on the surface and then weighed (W_n_) using electronic microbalance immediately. Swelling rate of the sample was calculated according to the following expression:

(6) $$\begin{array}{c} Swelling\;\;rate\left(\;\%\;\right)=\frac{W_{n}-W_{d}}{W_{d}}\times 100\% \end{array}$$

W_n_ is the weight of the swollen microspheres and W_d_ is the initial weight of the microspheres.

### 2.10. Determination of Tapped Density

The method of tapped densities was used according to the previous report [25]. The TD was obtained by measuring the weight of microspheres accurately and then putting in plastics pycnometer of 5 mL, after measuring the tapping was continued on a hard surface at a rate of 30 taps per min until no further change in volume was noted. The tapped density was expressed as follows:

(7) $$\begin{array}{c} Tapped\;\;density\left(\;\%\;\right)=\frac{W}{V}\times 100\% \end{array}$$

### 2.11. Determination of Moisture Contents

The moisture contents of the spray-dried microspheres were measured according to the guidelines of Chinese Pharmacopoeia (2010). Samples of approximately 100 mg were tested at a drying temperature of 105°C, until the difference of the successive two times of the weight was less than 0.3 mg. The moisture contents were obtained as the weight loss (%) and calculated as the mean ± SD from three independent measurements.

(8) $$\begin{array}{c} Moisture\;\;contents\left(\;\%\;\right)=\frac{W-W^{\prime }}{W}\times 100\% \end{array}$$

where W represents the mass of microspheres before tested and W′ represents the mass of microspheres after drying until the difference of the continuous two times of the weight is less than 0.3 mg.

### 2.12. Statistical Analysis

All experiments were performed three times, and the results are presented as mean ± SD. Statistical analysis was carried out with one-way ANOVA using SPSS19.0 and differences were considered to be significant at a level of P < 0.05.

## 3. Results and Discussion

### 3.1. Yield, Encapsulation Efficiency, and Drug Load

The YD, EE, and DL of PC/CTS/LEC microspheres with different ratios of drug carrier were determined by the above method and the results were shown in Table 2. Spray-dried process related dryness of PC was not a concerns, considering the knowledge that their stability is not compromised after this procedure, with a short period of exposure to heat. The microspheres had a high capacity in loading PC resulting from entrapping and adsorption. The different drug carrier ratios lead to the incoordination of EE and DL of five microspheres. With increasing the concentration of LEC in the formulation, EE and DL of microspheres increased. Since the numerous hydrophilic groups of LEC allow more water molecules moving to the inside of LEC, simultaneously PC was brought into LEC with water, such that the EE and DL of PC/CTS/LEC microspheres were elevated. On the contrary, by increasing the concentration of CTS in the formulation, EE and DL of microspheres decreased, due to the lower reacetylation of CTS [26]. From Table 2, it can be seen that as the proportion of drug/carrier increased, the DL of microspheres gradually increased. There are many factors affecting the yield of microspheres, including instrument specifications, instrument adhesion, dosage, and experimental operations. However, the inner wall of the deposition apparatus during the spraying of PC/CTS/LEC microspheres was less, and the resulting microspheres had a high YD.

### 3.2. Morphology of Microspheres

**Figure 1:**
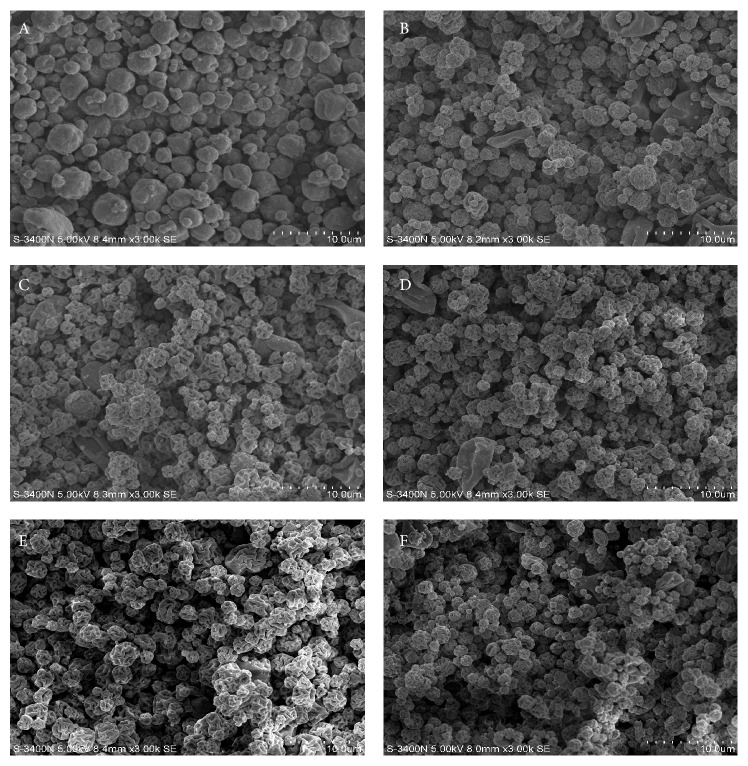
SEM microphotographs of spray-dried microspheres (×5000, Microspheres A: CTS:LEC = 1:1; Microspheres B: PC:CTS:LEC = 1:3:2; Microspheres C: PC:CTS:LEC = 1:1:1; Microspheres D: PC:CTS:LEC = 1:2:1; Microspheres E: PC:CTS:LEC = 1:1:2; Microspheres F: PC:CTS:LEC = 1:4:1).

The morphologies of PC/CTS/LEC microspheres are shown in Figure 1. Microspheres were prepared according to different formulations by using spray-drying method. Morphology represents an important parameter in microspheres intended for inhalation purposes, essentially considering aggregation, which may interfere with the flowing properties. The microspheres presented with spherical shape and were not aggregated. The blank microspheres had slightly wrinkled surfaces, while the PC/CTS/LEC microspheres possessed much wrinkled surface in this study. Some debris was produced in microspheres B, C, and D, which were unformed microspheres. The size of microspheres E was larger than that of F, and the dispersity of microspheres E was better than that of F. Hulse et al. [27] reported that the formation of wrinkled surface in morphology of microspheres may be caused by the excessive accumulation of vapor pressure during the evaporation process of the solvent during the spray-drying process. The surface-to-volume ratio of PC/CTS/LEC microspheres is increased because of the wrinkled surface, which could reduce the dose and frequency of drug delivery and thus increase the patient's compliance. Jensen et al. [28] reported that some degree of surface wrinkle is beneficial to the inhalation of microspheres, since it is reported that wrinkled microspheres can reduce the effects of the inhaler device and respiration [29]. Microspheres with wrinkled surface can increase the contact between the microspheres and the cell membrane at the site of absorption. In addition, the adhesion of CTS also increased the contact time between the microspheres and the cell membrane at the absorbed part, changed the fluidity of the cell membrane, prolonged the retention time of the microspheres in the gastrointestinal tract, and increased the penetrability of the drug into small intestinal epithelial cells [30]. This will promote the absorption of drugs and increase their bioavailability. The other important factor of the aerosol that deposits in the lungs is the diameters. The aerodynamic diameters of particles for optimal lung inhalation should be approximately 0.5~5 *μ*m. Larger than 6 *μ*m is generally deposited in the upper respiratory tract, and smaller than 0.5 *μ*m is exhaled without deposition [31]. The particle sizes of PC/CTS/LEC microspheres were between 0.5~5.0 *μ*m, such that the drug within this delivery system may deeply penetrate the lungs. Accordingly, the PC/CTS/LEC microspheres are fabricated by taking advantage of spray-drying method with much wrinkled surface and suitable diameters for lung inhalation, and they have a broad prospect as a drug carrier in the delivery of pulmonary medication.

### 3.3. FT-IR Study

**Figure 2:**
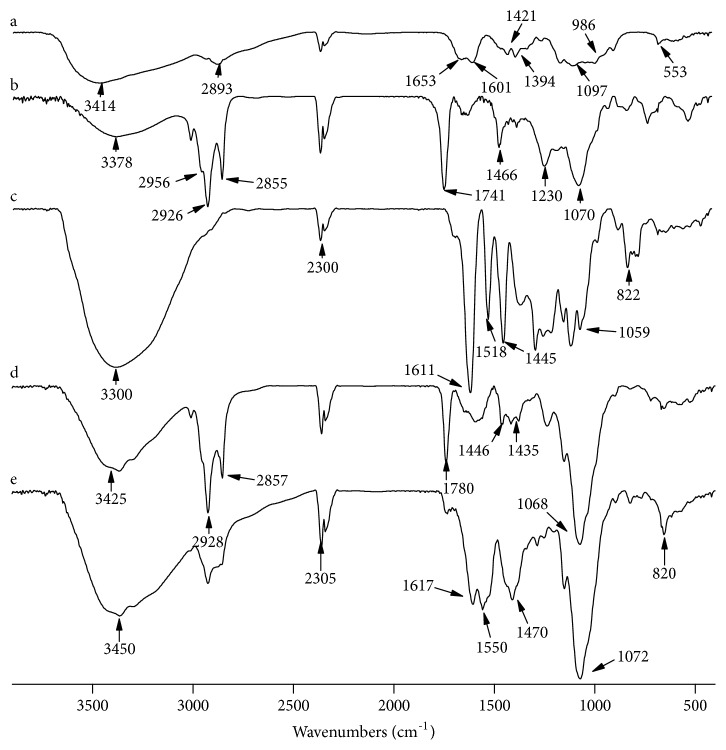
FT-IR spectra of the PC/CTS/LEC systems (a: CTS; b: LEC; c: PC; d: CTS/LEC microspheres; e: PC/CTS/LEC microspheres).

FT-IR spectra of CTS, LEC, PC, CTS/LEC microspheres, and PC/CTS/LEC microspheres (PC: CTS: LEC=1:1:2) are shown in Figure 2. The FT-IR spectrum of CTS showed a weak band of -OH stretching at 3400-3300 cm^−1^, the absorption band of the carbonyl (C=O) stretching of the secondary amide (amide I band) at 1653 cm^−1^, and the bending vibrations of the N-H (N-acetylated residues, amide II band) at 1601 cm^−1^. The peaks at 1421 and 1394 cm^−1^ belong to the N-H stretching of the amide, ether bonds, and N-H stretching (amide III band), respectively. The peaks observed at 1097 and 986 cm^−1^ were the secondary hydroxyl group (characteristic peak of –CH-OH in cyclic alcohols, C-O stretch) and the primary hydroxyl group (characteristic peak of -CH_2_-OH in primary alcohol, C-O stretch) (Figure 2a) [32]. The most prominent features of lecithin are bands corresponding to the hydrophobic tail regions at 2855, 2926, 2956, and 1466 cm^−1^. These represent symmetric CH_2_ (*υ*_s_CH_2_), antisymmetric CH_2_ (*υ*_as_CH_2_), antisymmetric CH_3_ (*υ*_as_CH_3_) stretching, and CH_2_ scissoring, respectively. The peak at 1741 cm^−1^ represents the C=O stretching (Figure 2b) [33]. This group is located in between hydrophobic tails and hydrophilic head group of the lecithin molecule. The spectrum of PC presents a single band at 1518 cm^−1^, and it is the characteristic peak of PC. Another characteristic display band of PC is at about 780~770 cm^−1^. The peaks observed at 1059 and 822 cm^−1^ were the hydroxyl group and C-O stretch (Figure 2c) [34]. The FT-IR spectra of the CTS/LEC microspheres (Figure 2d) showed that peaks at 1072 cm^−1^ were the hydroxyl group. The FT-IR spectra of the PC/CTS/LEC microspheres were similar except that the peaks of hydroxyl group shifted slightly toward lower wavenumbers to 1068 cm^−1^ and the peaks widening (Figure 2e).

The results displayed that there was no chemical interaction between PC and carrier materials, so PC was successfully embedded in CTS/LEC matrix through hydrogen bonding or transformed amorphous structure during the preparation of PC/CTS/LEC microspheres [35]. The main interaction between the CTS, LEC, and PC was electrostatic interaction and hydrogen bonding.

### 3.4. DSC Study

**Figure 3:**
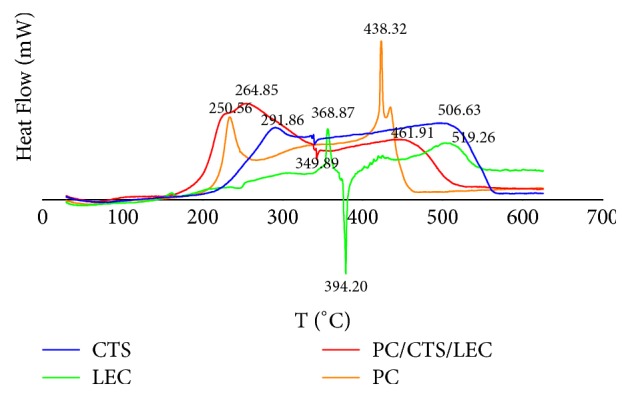
DSC thermograms of PC/CTS/LEC microspheres.

DSC thermograms of CTS, LEC, PC, and PC/CTS/LEC microspheres (PC: CTS: LEC = 1:1:2) are shown in Figure 3. The DSC thermogram of all the samples exhibited a broad endothermic peaks at about 100°C attributed to the volatilisation of associated water and the degradation of micromolecular. Pure CTS showed exothermic peaks at 291.86°C and 506.63°C, LEC showed exothermic peaks at 368.87°C and 519.26°C and an endothermic peak at 394.2°C, and PC had exothermic peaks at 250.56°C and 438.32°C. The markedly melting peak was found on CTS, LEC, and PC. These results suggested that CTS, LEC, and PC had the properties of crystals. The DSC of the PC-loaded microspheres displayed an endothermic peak at 349.89°C, and exothermic peaks at 264.85°C and 461.91°C where no characteristic peaks of PC was observed. The complete disappearance of the drug peak revealed that PC was embedded in the CTS/LEC microspheres. The results were consistent with the results of FT-IR. The samples were melting following crystallization and degradation at these endothermic peaks [36]. Our results indicated that PC existed in an amorphous or disordered crystalline phase as a molecular dispersion in polymeric matrix. Moreover, the physical state of the microspheres was studied because it had an important influence on the* in vitro* release characteristics. These results found that there was no interaction between the drug and other excipients, indicating that the drug may be slowly released in the body [37].

### 3.5. In Vitro Release

**Figure 4:**
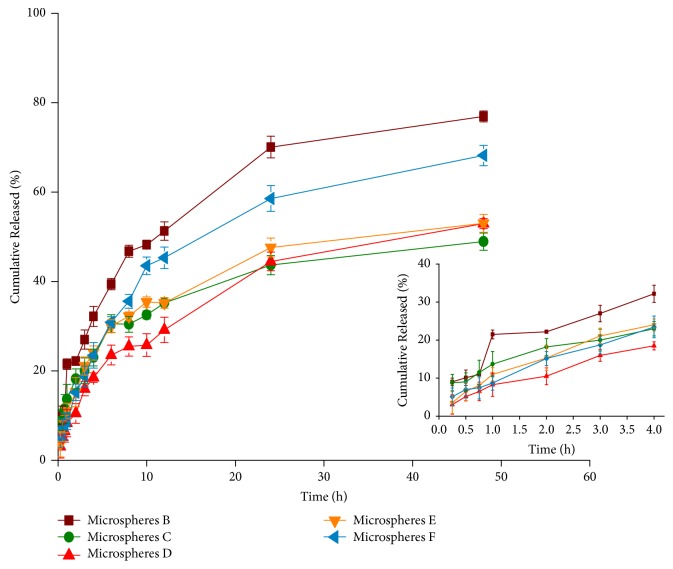
Release profile of PC from the spray-dried microspheres at PBS (pH 6.8, mean ± SD, n = 3).

In vitro release profiles of PC/CTS/LEC microspheres in PBS (pH 6.8) medium are shown in Figure 4. For microspheres B, C, D, E, and F, the PC released 24.24%, 17.72%, 11.60%, 17%, and 15.68% within 2 h, respectively. At the middle stage (24 h), it reached 70.08%, 43.68%, 44.48%, 47.60%, and 58.56%, respectively. The release rates of microspheres were up to 76.92%, 48.92%, 51.88%, 53.00%, and 68.2% within 48 h, respectively. A slow release was detected at initial time, and then a constant release rate from microspheres was observed, which could serve as a sustained release preparation for drug delivery system. The slow release might be ascribed to PC encapsulated into microspheres. PC release from microsphere was related to the concentration of CTS and the amount of PC. The PC release from microspheres slowed down significantly when the amount of PC increased and the concentration of CTS decreased in the formulations. Jiang et al. [38] reported that CTS affected drug release from microspheres may be due to the increasing amine groups and length of CTS chain in the particle structure. According to the previous study [39], the low-molecular-weight CTS-based fucospheres tended to weaken bonding and release drug. The influence of microspheres sustained release could be explained by the chain entanglement effect of CTS [40]. Furthermore, the drug/carrier ratio was an important factor influencing the release of microspheres. As the ratio of drug/carrier increased, the release of the drug was accelerated, which was mainly related to the concentration difference inside and outside the microsphere membrane and some pores generated during the release process. However, when the proportion of the carrier was increased to a certain extent, the sustained release effect did not increase. Only if the proportion of drug/carrier was appropriate, sustained release effect can be obtained. Zhang et al. [21] also found that theophylline/chitosan/*β*-cyclodextrin microspheres were similarly released by spray drying. Therefore, an ideal slow-release effect required a proper ratio of drug and carrier.

### 3.6. Moisture Contents Study

**Figure 5:**
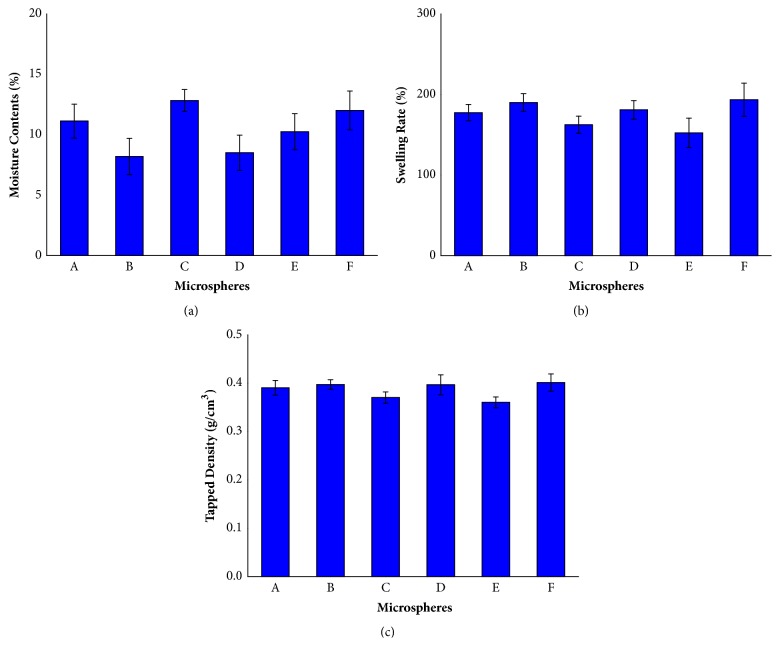
The moisture content, swelling rate, and tapped density of PC/CTS/LEC microspheres: (a) moisture content; (b) swelling rate; (c) tapped density.

The moisture contents of the blank and PC/CTS/LEC microspheres studied in this research are illustrated in Figure 5(a). As apparent from the figure, the moisture contents of microspheres range from 8% to 13%. The moisture contents of microspheres A, B, C, D, E, and F were lower than 13%, and there were no significant differences. Water is the medium of chemical reaction; thus, low moisture contents are highly desirable, since it will significantly avoid interparticulate cohesion and drug degradation and attain long-term stability [41]. Otherwise, the low moisture contents decreased the particle density and the aerodynamic characteristics were improved. By contrast, high moisture contents may negatively affect the stability of powders [42]. The moisture contents varied according to the inlet temperature, the outlet temperature, and the sample drying rate during the spray drying. A certain proportion of water content of the microspheres can reduce the water absorption and improve the stability of the microspheres.

### 3.7. Swelling Study

When contacting with aqueous medium, the PC/CTS/LEC microspheres were found to be capable of taking up water. The degree of swelling of microspheres generally depends on the properties of drug and polymeric material. The swelling rate is an important index closely related to the release of the encapsulated drug. Actually, plenty of water absorption leads to the dissolution of the microspheres and faster release of encapsulated drug [43]. The higher the swelling rate was, the easier the drugs release was. On the contrary, the lower the swelling rate was, the slower the drug release was. The results demonstrated that the swelling rate of microspheres E, C, A, D, B, and F increased, which were related to the concentration of CTS (Figure 5(b)). The higher the CTS was added, the larger the swelling rate of microspheres was. Rao et al. [44] reported that CTS can be facilely hydrated in water because it contains free hydroxyl and amino groups. However, after spray-dried with PC and LEC, the carbonyl group of LEC formed hydrogen bonds with the amide group of CTS, and the hydroxyl group of PC, the numbers of amino groups were decreased and thereby the hydrophilicity of the PC/CTS/LEC microspheres was changed [45]. Therefore, the swelling rate of the microspheres decreased, the drug release was slow, and the microspheres had a sustained release effect, which was consistent with the* in vitro* release results. Another study reported that the water absorption capacity of CTS was also related to the degree of cross-linking. The higher the degree of CTS cross-linking, the stronger the water absorption capacity [46].

### 3.8. Tapped Density Study

The results of the tapped density were shown in Figure 5(c). Tapped density was low without differences among all the formulations, varying between 0.35 and 0.4 g/cm.^3^ The results showed that the tapped density of microspheres E, C, D, B, A, and F increased and were related to the concentration of CTS. By increasing the concentration of CTS in the formulations, tapped density of PC/CTS/LEC microspheres increased. The tapped density is combining with the aerodynamic diameter and is an important parameter of microspheres to succeed in pulmonary delivery, especially for aggregation, dispersion, and deposition. According to our previous studies, spray-dried microspheres can be used in pulmonary delivery systems with satisfactory fluidity and dispersibility [18]. From the results of tapped density experiments, it can be observed that with the increase of the CTS ratio, the tapped density of the microspheres was greater, which may be due to the greater tapped density of CTS itself, so the proportion of CTS should be reduced as much as possible. Results were similar to those of previously pulmonary drug delivery system study [47].

### 3.9. Moisture Uptake

**Figure 6:**
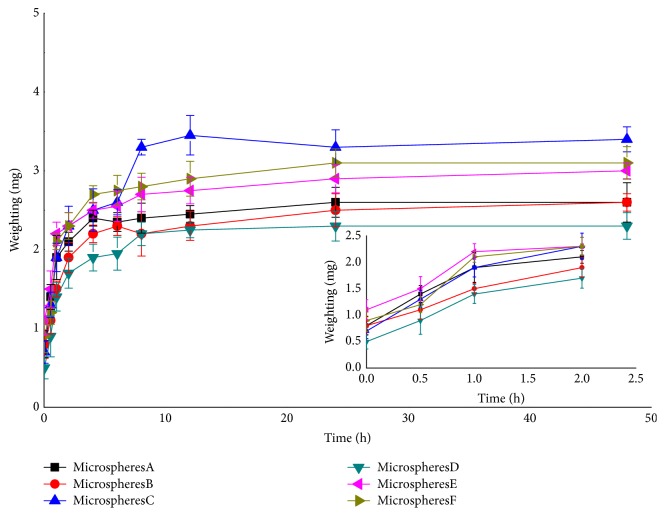
The moisture uptake of PC/CTS/LEC microspheres.

The increased weight of microspheres by moisture absorption could directly reflect the influence of relative humidity on powder. An increase in moisture of microspheres can influence the particle size, the powder aggregation, the dispersity, and the efficiency of inhalation. Accordingly, hydroscopicity is an important index of power. The moisture uptake of the blank and PC/CTS/LEC microspheres at 40°C and a relative humidity of 75% were studied and the moisture uptake profile of microspheres is shown in Figure 6. The moisture uptake of microspheres varied from 2.03 mg to 4.09 mg for 12 h, and this was followed by as low moisture uptake. The moisture uptake of microspheres with different drug-to-carrier ratio has no difference. The moisture absorption rate of the microspheres will affect the drug stability and sustained release effect, because moisture absorption can cause the microspheres to rupture and internal drug extravasation. This parameter was closely related to drug release, drug delivery, bioadhesive properties, and storage methods. These results indicated that the microspheres could absorb moisture at a temperature of 40°C/RH 75%. However, if the microspheres were stored in an inhaler, absorption may not occur. Considering that the humidity of the microspheres affects their stability and absorption* in vivo*, the microspheres should be stored at dry environment.

## 4. Conclusion

In this research, the PC/CTS/LEC microspheres were fabricated via using spray-drying method. The microspheres had a high EE and DL and were with a sustained release effect, which can reduce the frequency and dose of the drug, thus improving the compliance of the patients. The results of SEM revealed that the microspheres were spherical shape with more wrinkled surface, which could serve as a carrier for pulmonary drug delivery. The FT-IR spectrum demonstrated that the carbonyl group of LEC formed hydrogen bonds with the amide group of CTS and the hydroxyl group of PC. DSC study displayed that the stability of microspheres was improved. Furthermore, the PC/CTS/LEC microspheres possessed desirable moisture contents, swelling rate, tapped density, and moisture uptake, and preferable stability therefore would be effective as pulmonary drug carriers.

## Figures and Tables

**Table 1:** Composition of formulations used for aqueous solution to be spray dried.

Microspheres	PC (%, W/V), (g)	CTS (%, W/V), (g)	LEC (%, W/V), (g)	PC/CTS/LEC
A	/	1 (10)	1 (10)	1: 1
B	1 (10)	3 (30)	2 (20)	1: 3: 2
C	1 (10)	1 (10)	1 (10)	1: 1: 1
D	1 (10)	2 (20)	1 (10)	1: 2: 1
E	1 (10)	1 (10)	2 (20)	1: 1: 2
F	1 (10)	4 (40)	1 (10)	1: 4: 1

**Table 2:** The results of yield, drug loading, and encapsulation efficiency (mean ± SD, n = 3, %).

Microspheres	B	C	D	E	F
Yield	56.77 ± 1.46	60.57 ± 1.11	56.22 ± 0.98	44.23 ± 0.87	55.59 ± 1.11
Drug Loading	7.83 ± 0.21	16.83 ± 0.46	9.17 ± 0.22	16.96 ± 0.09	7.19 ± 0.09
Encapsulation Efficiency	46.98 ± 1.27	50.47 ± 1.35	36.66 ± 0.88	67.83 ± 0.36	43.12 ± 0.54

## Data Availability

The any data used to support the findings of this study are available from the corresponding author upon request.
